# Neutrophil to lymphocyte ratio predicts prognosis in unresectable pancreatic cancer

**DOI:** 10.1038/s41598-020-75745-8

**Published:** 2020-10-30

**Authors:** Naoto Iwai, Takashi Okuda, Junichi Sakagami, Taishi Harada, Tomoya Ohara, Masashi Taniguchi, Hiroaki Sakai, Kohei Oka, Tasuku Hara, Toshifumi Tsuji, Toshiyuki Komaki, Keizo Kagawa, Hiroaki Yasuda, Yuji Naito, Yoshito Itoh

**Affiliations:** 1 Department of Gastroenterology and Hepatology, Fukuchiyama City Hospital, 231 Atsunaka-cho, Fukuchiyama-city, Kyoto 620-8505 Japan; 2 Department of Molecular Gastroenterology and Hepatology, Graduate School of Medical Science, Kyoto Prefectural University of Medicine, Kyoto, Japan; 3 Department of Medical Oncology, Fukuchiyama City Hospital, Fukuchiyama-city, Kyoto Japan

**Keywords:** Cancer, Biomarkers, Gastroenterology, Oncology

## Abstract

Inflammation-based prognostic indicators have been developed to predict the prognosis in patients with pancreatic cancer. However, prognostic indices have not been established in patients with unresectable pancreatic cancer, including those without indication for chemotherapy at diagnosis. This study aimed to identify the predictors in all patients with unresectable pancreatic cancer. We retrospectively analyzed data of 119 patients with unresectable pancreatic cancer from June 2006 to September 2018. The following laboratory parameters were evaluated: the Glasgow Prognostic Score (GPS), modified GPS, neutrophil-to-lymphocyte ratio (NLR), platelet-to-lymphocyte ratio (PLR), C-reactive protein albumin (CRP/Alb) ratio, and prognostic nutritional index (PNI). We performed time-dependent receiver operating characteristic analysis, overall survival (OS) analysis, and univariate and multivariate analyses to determine the prognostic factors in patients with unresectable pancreatic cancer. The cut-off value for NLR was determined to be 3.74. The 6-month OS rates in low and high NLR groups were 75.5% and 18.8% (*P* < 0.001). In the univariate analysis, advanced age (*P* = 0.003), metastatic pancreatic cancer (*P* = 0.037), no treatment (*P* < 0.001), worse Eastern Cooperative Oncology Group Performance Status (ECOG-PS) (*P* < 0.001), high GPS (*P* < 0.001), high modified GPS (*P* < 0.001), high NLR (*P* < 0.001), high PLR (*P* = 0.002), high CRP/Alb ratio (*P* < 0.001), and low PNI (*P* < 0.001) were identified as the prognostic factors. The multivariate analysis revealed that metastatic pancreatic cancer (*P* = 0.046), no treatment (*P* < 0.001), worse ECOG-PS (*P* = 0.002), and high NLR (*P* < 0.001) were independently associated with OS. We revealed that the high NLR could be an independent indicator of poor prognosis in patients with unresectable pancreatic cancer.

## Introduction

Pancreatic cancer is one of the most aggressive types, with 5-year overall survival (OS) rate < 10%^1,2^. Due to the difficulty in early detection, most patients present with unresectable disease, and less than 30% of patients have resectable tumors when diagnosed^3^. Intensive chemotherapy regimens such as FOLFIRINOX and Gem plus nab-paclitaxel has been developed^4,5^, however, the median OS was 8.5–11.1 months in these clinical trials.

Recently, inflammation has been reported to be closely related to carcinogenesis and progression of pancreatic cancer^6^. In clinical settings, systemic inflammation-based prognostic indicators have been developed to predict the prognosis in patients with pancreatic cancer. Among laboratory parameters, the modified Glasgow Prognostic Score (GPS)^7,8^, neutrophil-to-lymphocyte ratio (NLR)^9–13^, platelet-to-lymphocyte ratio (PLR)^14^, C-reactive protein albumin (CRP/Alb) ratio^15,16^, and prognostic nutritional index (PNI)^17^, have been reported to be predictive factors of OS in patients with pancreatic cancer. The subjects in most of the previous studies were the patients who underwent surgical resection or chemotherapy. However, in a real-world setting, some patients with unresectable pancreatic cancer have no indication for chemotherapy at diagnosis because of advanced age, or worse, their general condition. Thus, it is important to establish precise prognostic biomarkers in all patients with unresectable pancreatic cancer.

In this study, our goal is to identify the prognostic predictors in patients with unresectable pancreatic cancer, and to evaluate the corresponding laboratory parameters. To this end, we retrospectively evaluated the patient laboratory parameters.

## Results

### Patient characteristics

Table 1 shows the characteristics of the 119 patients in this study. The data for 119 patients with unresectable pancreatic cancer were evaluated. The median age was 73 years old (range 43–94), and median follow-up day was 136 days (range 4–1252). During follow-up, 116 patients died. Of the enrolled patients, 52.9% were male, and 28.6% had Eastern Cooperative Oncology Group Performance Status (ECOG-PS) ≥ 2. Regarding the tumor lesion, 40.3% were located in the head, and 88.2% were diagnosed as metastatic pancreatic cancer. Regarding the treatment, 31.9% received best supportive care (BSC), 7.6% underwent chemoradiotherapy, and 60.5% underwent chemotherapy. In patients receiving BSC, the median age was 82.0 years old (range 53–94), and 36.8% opted for BSC because of the advanced age itself.

**Table 1:** Patient characteristics.

No. of patients	119	
Age (years), median (range)	73	(43–94)
Follow-up (day), median (range)	136	(4–1252)
Total of deaths	116	
Body mass index, median (range)	20.56	(15.11–30.25)
**Gender, n (%)**		
Male	63	(52.9)
Female	56	(47.1)
**ECOG-PS, n (%)**		
< 1	84	(70.6)
≥2	34	(28.6)
Unknown	1	(0.8)
**Location, n (%)**		
Head	48	(40.3)
Body or tail	71	(59.7)
**Stage, n (%)**		
Locally advanced	14	(11.8)
Metastatic	105	(88.2)
**Treatment, n (%)**		
Best Supportive Care	38	(31.9)
Chemoradiotherapy	9	(7.6)
Chemotherapy	72	(60.5)
FOLFIRINOX	18	(15.1)
Gem + nabPTX	6	(5.0)
Gem + S1	7	(5.9)
Gem + elrotinib	1	(0.8)
Gem	40	(33.6)
GPS, (0:1:2:unknown)	42:42:33:2	
modified GPS, (0:1:2:unknown)	50:34:33:2	
NLR, median (range)	4.32	(1.00–39.97)
PLR, median (range)	179.40	(38.50–655.41)
CRP/Alb ratio	0.41	(0.004–12.34)
PNI median, (range)	43.44	(24.25–61.96)

*ECOG-PS* Eastern Cooperative Oncology Group Performance Status; *GPS* Glasgow Prognostic Score; *NLR* neutrophil-to-lymphocyte ratio; *PLR* platelet-to-lymphocyte ratio; *CRP* C-reactive protein; *Alb* albumin; *PNI* prognostic nutritional index.

### NLR is a useful prognostic marker in patients with unresectable pancreatic cancer

**Figure 1:**
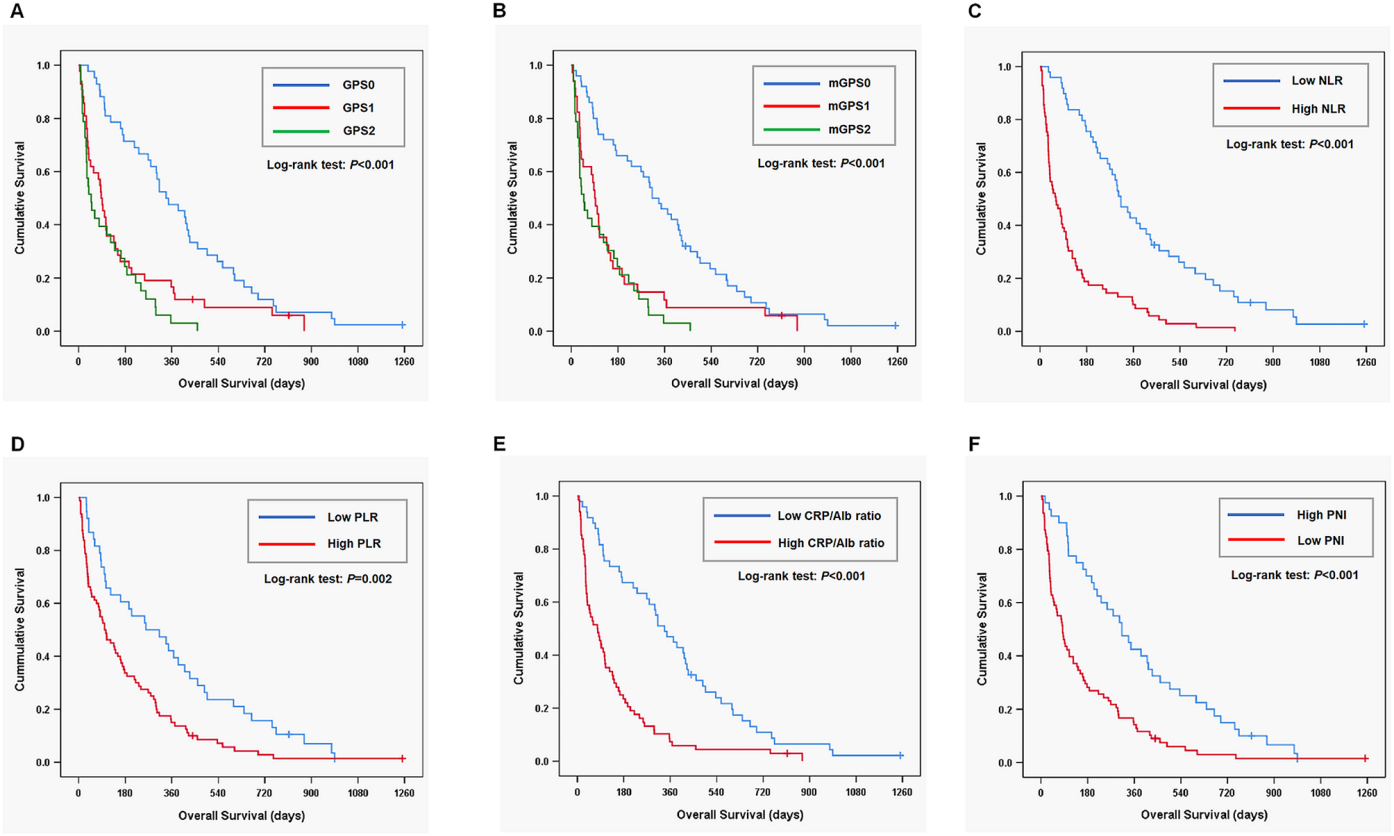
The overall survival (OS) according to prognostic factors in patients with unresectable pancreatic cancer. *GPS* Glasgow Prognostic Score, *NLR* neutrophil to lymphocyte ratio, *PLR* platelet to lymphocyte ratio, *CRP/Alb ratio* C-reactive protein albumin ratio, *PNI* prognostic nutritional index.

Table 2 shows the area under the curve (AUC) for OS variables using time-dependent receiver operating characteristic (ROC) curve at the 6-month follow-up. NLR had the highest value (0.792) among the prognostic factors. Figure 1A–F show the relationship between the prognostic factors and OS. The cut-off values for NLR, PLR, CRP/Alb ratio, and PNI were determined as 3.74, 146, 0.28, and 46.8, respectively. A lower GPS, modified GPS, NLR, PLR, and CRP/Alb ratio were significantly associated with a higher OS. While, a higher PNI was closely related to a higher OS (*P* < 0.001). The six-month OS rates in GPS0, GPS1, and GPS2 subgroups were 71.4%, 26.2%, and 24.2%, respectively, (*P* < 0.001; Fig. 1A), while those in the modified GPS0, modified GPS1, and modified GPS2 were 66.0%, 23.5%, and 24.2%, respectively, (*P* < 0.001; Fig. 1B). The 6-month OS rates in the low and high NLR groups were 75.5% and 18.8%, respectively, (*P* < 0.001; Fig. 1C), while those in the low and high PLR groups were 60.5% and 33.7%, respectively, (*P* = 0.002; Fig. 1D). The 6-month OS rates in the low and high CRP/Alb groups were 67.3% and 23.5%, respectively, (*P* < 0.001; Fig. 1E), while those in the high and low PNI groups were 70.0% and 28.2%, respectively, (*P* < 0.001; Fig. 1F).

**Table 2:** AUC in variables for overall survival at 6-month follow-up.

	AUC	95% CI	*P value*
GPS	0.722	0.627–0.809	< 0.001
modified GPS	0.711	0.618–0.797	< 0.001
NLR	0.792	0.705–0.870	< 0.001
PLR	0.631	0.531–0.731	0.016
CRP/Alb ratio	0.753	0.661–0.839	< 0.001
PNI	0.719	0.623–0.806	< 0.001

*AUC* area under the curve; *CI* confidence interval; *GPS* Glasgow Prognostic Score; *NLR* neutrophil-to-lymphocyte ratio; *PLR* platelet-to-lymphocyte ratio; *CRP* C-reactive protein; *Alb* albumin; *PNI* prognostic nutritional index.

### High NLR is independently associated with OS

Table 3 shows univariate and multivariate analyses of the predictive factors of OS in patients with unresectable pancreatic cancer. In the univariate analysis, advanced age (hazard ratio [HR] = 1.035; 95% confidence interval [CI] 1.012–1.058; *P* = 0.003), metastatic pancreatic cancer (HR 1.819; 95% CI 1.036–3.196; *P* = 0.037), no treatment (HR 4.632; 95% CI 2.971–7.222; *P* < 0.001), worse ECOG-PS (HR 4.309; 95% CI 2.726–6.812; *P* < 0.001), GPS 2/1 (HR 3.637; 95% CI 2.215–5.973; *P* < 0.001/HR 2.228; 95% CI 1.424–3.487; *P* < 0.001), modified GPS 2/1 (HR 3.357; 95% CI 2.082–5.411; *P* < 0.001/HR 2.167; 95% CI 1.373–3.421; *P* = 0.001), high NLR (HR 3.363; 95% CI 2.251–5.025; *P* < 0.001), high PLR (HR 1.878; 95% CI 1.256–2.807; *P* = 0.002), high CRP/Alb ratio (HR 2.674; 95% CI 1.803–3.966; *P* < 0.001), and low PNI (HR 2.251; 95% CI 1.511–3.353; *P* < 0.001) were identified as prognostic factors. The multivariate analysis revealed that metastatic pancreatic cancer (HR 1.923; 95% CI 1.013–3.649; *P* = 0.046), no treatment (HR 5.635; 95% CI 3.076–10.325; *P* < 0.001), worse ECOG-PS (HR 2.466; 95% CI 1.410–4.313; *P* = 0.002), and high NLR (HR 2.430; 95% CI 1.484–3.977; *P* < 0.001) were independently associated with OS.

**Table 3:** Univariate and multivariate analyses of prognostic factors in patients with unresectable pancreatic cancer.

	Univariate analysis			Multivariate analysis		
	HR	95% CI	*P value*	HR	95% CI	*P value*
**Age**						
Per year	1.035	1.012‐1.058	0.003			
**Gender**						
Female	1					
Male	1.004	0.692‐1.457	0.982			
**Tumor location**						
Head	1					
Body or tail	1.418	0.975‐2.062	0.068			
**cStage**						
Locally advanced	1			1		
Metastatic	1.819	1.036‐3.196	0.037	1.923	1.013‐3.649	0.046
**Treatment**						
Present	1			1		
Absent	4.632	2.971‐7.222	< 0.001	5.635	3.076‐10.325	< 0.001
**ECOG-PS**						
≤1	1			1		
> 1	4.309	2.726–6.812	< 0.001	2.466	1.410–4.313	0.002
**GPS**						
0	1					
1	2.228	1.424–3.487	< 0.001			
2	3.637	2.215–5.973	< 0.001			
**Modified GPS**						
0	1					
1	2.167	1.373–3.421	0.001			
2	3.357	2.082–5.411	< 0.001			
**NLR**						
≤3.74	1			1		
> 3.74	3.363	2.251–5.025	< 0.001	2.430	1.484–3.977	< 0.001
**PLR**						
≤146	1					
> 146	1.878	1.256–2.807	0.002			
**CRP/Alb ratio**						
≤0.28	1					
> 0.28	2.674	1.803–3.966	< 0.001			
**PNI**						
> 46.8	1					
≤46.8	2.251	1.511–3.353	< 0.001			

*HR* hazard ratio; *CI* confidence interval; *ECOG-PS* eastern cooperative oncology group; *GPS* Glasgow Prognostic Score; *NLR* neutrophil-to-lymphocyte ratio; *PLR* platelet-to-lymphocyte ratio; *CRP* C-reactive protein; *Alb* albumin; *PNI* prognostic nutritional index.

### High NLR is associated with the metastatic stage, and worse performance status

Table 4 shows clinical parameters in relation to NLR. Patients in the higher NLR group were significantly associated with metastatic pancreatic cancer (*P* = 0.001). ECOG-PS was significantly higher in patients in the higher NLR group (*P* < 0.001). GPS, modified GPS, PLR, and CRP/Alb ratio were significantly higher in the higher NLR group (*P* < 0.001), while PNI was significantly lower in the higher NLR group (*P* < 0.001).

**Table 4:** Clinical parameters in relation to NLR.

	NLR ≤ 3.74 (n = 49)	NLR > 3.74 (n = 69)	*P value*
Age, median (range)	72 (46–93)	74 (53–94)	0.512
Gender (female/male)	25/24	31/38	0.641
Tumor location (head/body or tail)	24/25	24/45	0.175
cStage (locally advanced/metastatic)	12/37	2/67	0.001
Treatment (present/absent)	38/11	43/26	0.120
ECOG-PS (≤ 1/ > 1)	44/5	40/29	< 0.001
GPS (0/1/2)	30/12/6	12/30/27	< 0.001
Modified GPS (0/1/2)	34/8/6	16/26/27	< 0.001
PLR, median (range)	128.10 (38.50–267.98)	202.01 (60.96–655.41)	< 0.001
CRP/Alb ratio, median (range)	0.06 (0.00–2.39)	0.87 (0.03–12.34)	< 0.001
PNI, median (range)	48.35 (34.60–61.96)	39.52 (24.25–51.51)	< 0.001

*NLR* neutrophil-to-lymphocyte ratio; *GPS* Glasgow Prognostic Score; *PLR* platelet-to-lymphocyte ratio; *CRP* C-reactive protein; *Alb* albumin; *PNI* prognostic nutritional index.

## Discussion

In this study, we evaluated the prognostic factors in patients with unresectable pancreatic cancer, including those without indication for chemotherapy. This study revealed that the NLR was an independent prognostic factor in patients with unresectable pancreatic cancer, and superior to the GPS, modified GPS, PLR, CRP/Alb ratio, and PNI as the prognostic indicators.

Inflammation has been recently considered to play an essential role in cancer progression. Moreover, inflammation-based prognostic factors have been developed such as the GPS^18^, modified GPS^19^, PLR^14^, CRP/Alb ratio^20^, and NLR^21^. NLR was originally established as the parameter of stress and systemic inflammation in clinical ICU practice^22^. Recently, NLR has been increasingly appreciated as a pivotal prognostic factor in various cancers^23,24^. With regard to the pancreatic cancer, previous studies revealed that NLR was a significant prognostic marker among various disease stages^9–13,25–30^. In resectable pancreatic cancer, previous studies have identified preoperative NLR as a useful prognostic marker^9,13^. In particular, Stotz et al., using a multivariate Cox proportional-hazard model in their study, reported that advanced tumor stage and high NLR (NLR > 5) were independent prognostic factors for operable pancreatic cancers^9^. In unresectable pancreatic cancer, most of the previous studies investigated the role of NLR in patients undergoing chemoradiotherapy or chemotherapy^10–12,25–27^. Some studies have illustrated that baseline NLR and post-chemotherapy changes in NLR values could predict OS in patients undergoing chemotherapy^12,26^. However, few have evaluated the prognosis in patients with advanced pancreatic cancer, including those without treatment^9^. In this study, NLR was shown to be an independent prognostic index in patients with unresectable pancreatic cancer, both with and without treatment. In an increasingly aging society, it is possible that among patients without an indication for chemotherapy at the time of diagnosis, there would be an increasing number of those with unresectable pancreatic cancer. Indeed, more than 30% of the enrolled patients received BSC in this study. In addition, the median age was more than 80 years old in patients receiving BSC. Collectively, NLR at the time of diagnosis could be useful for prognosis prediction in unresectable pancreatic cancer in our aging society.

In this study, we revealed that PNI could also predict the prognosis as well as the inflammation-based prognostic factors in the log-rank test and univariate analysis. PNI was originally established as the surgical risk indicator in patients undergoing gastrointestinal surgery^31^. Recently, PNI was reported to be useful in predicting the prognosis in the patients undergoing surgery for pancreatic cancer^17,32^. Our results suggested that nutritional status may be related to prognosis in patients with unresectable pancreatic cancer. Therefore, nutritional intervention may contribute to improving prognosis in unresectable pancreatic cancer.

In this study, NLR was found to be superior to the other factors tested in the multivariate and time-dependent ROC curve analyses. The cut-off value of the NLR level was 3.74 in this study, which is consistent with the level that ranged from 2.5 to 5.0 in the previous studies^9–12,25,26^. The mechanism of the relationship between NLR and prognosis in patients with unresectable pancreatic cancer remains to be clarified. Neutrophils inhibit the immune response by lymphocytes, natural killer cells, or activated T cells^33,34^, while lymphocytes reflect the immune response of the host to either infection or cancer. In addition, tumor-infiltrating lymphocytes are associated with better prognosis in patients with pancreatic ductal adenocarcinoma^35^. This study revealed that a high NLR was significantly associated with the metastatic stage, and worse performance status. Collectively, our results may suggest that NLR reflects both the disease progression and patient condition in unresectable pancreatic cancer.

There are some limitations in this study. First, it is a retrospective and a single-center study with small number of patients. Therefore, a multicenter prospective validation is needed to validate our results. Second, we defined the cut-off value for NLR as 3.74, although the cut-off values for NLR vary from 2.5 to 5.0 in unresectable pancreatic cancer^9–12,25,27^. The ideal cut-off value should be confirmed.

In conclusion, our results reveal that high NLR at the time of diagnosis could be an independent indicator of poor prognosis in patients with unresectable pancreatic cancer. Our findings suggest that a controlling factor for NLR could provide a novel therapeutic target for unresectable pancreatic cancer in the near future.

## Methods

### Study population

We retrospectively recruited 166 patients who had been diagnosed with pancreatic cancer from June 2006 to September 2018 at Fukuchiyama City Hospital. Of these 166 patients, we excluded 42 with resectable pancreatic cancer and 5 with borderline-resectable pancreatic cancer. Then, the data of a total of 119 patients with unresectable pancreatic cancer were analyzed in this study. The opt-out method was performed for obtaining informed consent due to the retrospective design. This retrospective study was consistent with the standards of the Declaration of Helsinki. This study was approved by the institutional review board of Fukuchiyama City Hospital (approval number: 1–44).

We collected patient clinical data on the age, body mass index, gender, tumor location, clinical stage, treatment, and prognoses. Clinical stage and their resectability criteria were determined based on the 7th edition of the Japan Pancreas Society guideline^36^. In addition, we assessed ECOG-PS and the laboratory parameters at the time of diagnosis. Laboratory parameters, including the GPS, the modified GPS, NLR, PLR, CRP/Alb ratio, and PNI were evaluated using blood samples. Data on prognoses were confirmed by medical record review from October 2019 to March 2020.

For the GPS, the modified GPS, NLR, PLR, CRP/Alb ratio, and PNI values, we performed time-dependent ROC analysis, and calculated AUC for OS at the 6-month of follow-up. Subsequently, the cut-off values for continuous variables were determined using the Youden’s Index. OS was defined as the time from the date of diagnosis to the date of death or last follow-up. OS was evaluated between the two groups divided by the cut-off values in each prognostic score.

Univariate and multivariate analyses were used to determine the predictive factors of OS in patients with unresectable pancreatic cancer. The candidate factors analyzed were as follows; age, gender, tumor location, clinical stage, treatment, ECOG-PS, GPS, modified GPS, NLR, PLR, CRP/Alb ratio, and PNI. Finally, we assessed the relation between NLR and other clinical parameters. The parameters were as follows; age, gender, tumor location, clinical stage, treatment, ECOG-PS, GPS, modified GPS, PLR, CRP/Alb ratio, and PNI.

### Statistical analysis

Data are shown as median and range. Time-dependent ROC analysis was conducted to determine the cut-off values for continuous variables. The OS rates were evaluated using Kaplan–Meier’s survival curves, and the log rank analysis was conducted to verify the significance of differences. Cox proportional hazards model analysis was conducted to calculate HR and 95% CI in the candidates of prognostic factors. A *P* value < 0.05 was statistically considered significant. All statistical analyses were performed using SPSS statistics version 26.0 (IBM Japan, Tokyo, Japan) and R software version 3.6.2.

## References

[1] Tuveson DA, Neoptolemos JP (2012). Understanding metastasis in pancreatic cancer: a call for new clinical approaches. Cell.

[2] Ilic M, Ilic I (2016). Epidemiology of pancreatic cancer. World J. Gastroenterol..

[3] Furuse J, Shibahara J, Sugiyama M (2018). Development of chemotherapy and significance of conversion surgery after chemotherapy in unresectable pancreatic cancer. J. Hepatobiliary Pancreat. Sci..

[4] Conroy T (2011). FOLFIRINOX versus gemcitabine for metastatic pancreatic cancer. N. Engl. J. Med..

[5] Von Hoff DD (2013). Increased survival in pancreatic cancer with nab-paclitaxel plus gemcitabine. N. Engl. J. Med..

[6] Perusina Lanfranca M (2020). Interleukin 22 signaling regulates acinar cell plasticity to promote pancreatic tumor development in mice. Gastroenterology.

[7] Jamieson NB (2011). A prospective comparison of the prognostic value of tumor- and patient-related factors in patients undergoing potentially curative surgery for pancreatic ductal adenocarcinoma. Ann. Surg. Oncol..

[8] La Torre M (2012). The glasgow prognostic score as a predictor of survival in patients with potentially resectable pancreatic adenocarcinoma. Ann. Surg. Oncol..

[9] Stotz M (2013). Increased neutrophil-lymphocyte ratio is a poor prognostic factor in patients with primary operable and inoperable pancreatic cancer. Br. J. Cancer.

[10] An X (2010). Elevated neutrophil to lymphocyte ratio predicts survival in advanced pancreatic cancer. Biomarkers.

[11] Hasegawa S (2016). Pre-treatment neutrophil to lymphocyte ratio as a predictive marker for pathological response to preoperative chemoradiotherapy in pancreatic cancer. Oncol. Lett..

[12] Chen Y, Yan H, Wang Y, Shi Y, Dai G (2017). Significance of baseline and change in neutrophil-to-lymphocyte ratio in predicting prognosis: a retrospective analysis in advanced pancreatic ductal adenocarcinoma. Sci. Rep..

[13] Bhatti I, Peacock O, Lloyd G, Larvin M, Hall RI (2010). Preoperative hematologic markers as independent predictors of prognosis in resected pancreatic ductal adenocarcinoma: neutrophil-lymphocyte versus platelet-lymphocyte ratio. Am. J. Surg..

[14] Smith RA (2009). Preoperative platelet-lymphocyte ratio is an independent significant prognostic marker in resected pancreatic ductal adenocarcinoma. Am. J. Surg..

[15] Haruki K (2016). The C-reactive protein to albumin ratio predicts long-term outcomes in patients with pancreatic cancer after pancreatic resection. World J. Surg..

[16] Wu M, Guo J, Guo L, Zuo Q (2016). The C-reactive protein/albumin ratio predicts overall survival of patients with advanced pancreatic cancer. Tumour Biol..

[17] Ikeguchi M (2019). Clinical importance of preoperative and postoperative prognostic nutritional index in patients with pancreatic ductal adenocarcinoma. Ann. Hepatobiliary. Pancreat. Surg..

[18] Forrest LM, McMillan DC, McArdle CS, Angerson WJ, Dunlop DJ (2004). Comparison of an inflammation-based prognostic score (GPS) with performance status (ECOG) in patients receiving platinum-based chemotherapy for inoperable non-small-cell lung cancer. Br. J. Cancer.

[19] Toiyama Y (2011). Evaluation of an inflammation-based prognostic score for the identification of patients requiring postoperative adjuvant chemotherapy for stage II colorectal cancer. Exp. Ther. Med..

[20] Fairclough E, Cairns E, Hamilton J, Kelly C (2009). Evaluation of a modified early warning system for acute medical admissions and comparison with C-reactive protein/albumin ratio as a predictor of patient outcome. Clin. Med. (Lond., Engl.).

[21] Halazun KJ (2009). Negative impact of neutrophil-lymphocyte ratio on outcome after liver transplantation for hepatocellular carcinoma. Ann. Surg..

[22] Zahorec R (2001). Ratio of neutrophil to lymphocyte counts–rapid and simple parameter of systemic inflammation and stress in critically ill. Bratisl Lek Listy..

[23] Guthrie GJ (2013). The systemic inflammation-based neutrophil-lymphocyte ratio: experience in patients with cancer. Crit. Rev. Oncol. Hematol..

[24] Templeton AJ (2014). Prognostic role of neutrophil-to-lymphocyte ratio in solid tumors: a systematic review and meta-analysis. J. Natl. Cancer Inst..

[25] Wang DS (2012). Comparison of the prognostic values of various inflammation based factors in patients with pancreatic cancer. Med. Oncol..

[26] Luo G (2015). Blood neutrophil-lymphocyte ratio predicts survival in patients with advanced pancreatic cancer treated with chemotherapy. Ann. Surg. Oncol..

[27] Lee BM, Chung SY, Chang JS, Lee KJ, Seong J (2018). The neutrophil-lymphocyte ratio and platelet-lymphocyte ratio are prognostic factors in patients with locally advanced pancreatic cancer treated with chemoradiotherapy. Gut Liver.

[28] Pu N (2019). Independent effect of postoperative neutrophil-to-lymphocyte ratio on the survival of pancreatic ductal adenocarcinoma with open distal pancreatosplenectomy and its nomogram-based prediction. J. Cancer..

[29] Zhou Y (2018). Prognostic role of the neutrophil-to-lymphocyte ratio in pancreatic cancer: a meta-analysis containing 8252 patients. Clin. Chim. Acta..

[30] Mowbray NG (2018). A meta-analysis of the utility of the neutrophil-to-lymphocyte ratio in predicting survival after pancreatic cancer resection. HPB (Oxford)..

[31] Onodera T, Goseki N, Kosaki G (1984). Prognostic nutritional index in gastrointestinal surgery of malnourished cancer patients. Nihon Geka Gakkai Zasshi..

[32] Kanda M (2011). Nutritional predictors of postoperative outcome in pancreatic cancer. Br. J. Surg..

[33] Petrie HT, Klassen LW, Kay HD (1985). Inhibition of human cytotoxic T lymphocyte activity in vitro by autologous peripheral blood granulocytes. J. Immunol..

[34] El-Hag A, Clark RA (1987). Immunosuppression by activated human neutrophils. Dependence on the myeloperoxidase system. J. Immunol..

[35] Lianyuan T, Dianrong X, Chunhui Y, Zhaolai M, Bin J (2018). The predictive value and role of stromal tumor-infiltrating lymphocytes in pancreatic ductal adenocarcinoma (PDAC). Cancer Biol. Ther..

[36] Japan Pancreas Society (2016). General Rules for the Study of Pancreatic Cancer.

